# Spatial Electron-hole Separation in a One Dimensional Hybrid Organic–Inorganic Lead Iodide

**DOI:** 10.1038/srep20626

**Published:** 2016-02-09

**Authors:** Christopher N. Savory, Robert G. Palgrave, Hugo Bronstein, David O. Scanlon

**Affiliations:** 1 University College London, Kathleen Lonsdale Materials Chemistry, Department of Chemistry, 20 Gordon Street, London WC1H 0AJ, UK; 2 University College London, Department of Chemistry, London WC1H 0AJ, UK; 3 Diamond Light Source Ltd., Diamond House, Harwell Science and Innovation Campus, Didcot, Oxfordshire OX11 0DE, UK

## Abstract

The increasing efficiency of the inorganic-organic hybrid halides has revolutionised photovoltaic research. Despite this rapid progress, the significant issues of poor stability and toxicity have yet to be suitably overcome. In this article, we use Density Functional Theory to examine (Pb_2_I_6_) · (H_2_DPNDI) · (H_2_O) · (NMP), an alternative lead-based hybrid inorganic-organic solar absorber based on a photoactive organic cation. Our results demonstrate that optical properties suitable for photovoltaic applications, in addition to spatial electron-hole separation, are possible but efficient charge transport may be a limiting factor.

The total energy consumption of the EU is set to double by 2050, and so the necessary expansion of renewable energy resources has become readily apparent – the demand for photovoltaic cells has increased rapidly over the last two decades^1^,^2^. Currently, the photovoltaic market is dominated by crystalline silicon wafer solar cells, but with high energetic and monetary cost for producing such modules, there is a pressing need to develop materials that can provide equally efficient cells yet a lower manufacturing cost and pay-back time^3^,^4^. While thin film materials, such as Cu(In, Ga)Se_2_, have seen high power conversion efficiencies (PCEs) in the laboratory^5^, these efforts have been hampered by recombination losses^6^ and the relatively low abundance of indium and gallium, leading to their corresponding high costs^1^.

A promising family of materials in the field of earth abundant thin film solar cells are the methylammonium lead halides, CH_3_NH_3_PbX_3_ (X = Cl, Br, I), which after being introduced as photoabsorbers in dye-sensitized solar cells (DSSCs) in 2009^7^, have seen enormous interest from the scientific community^8^,^9^,^10^. With reported PCEs nearing or exceeding 20% (comparable to records for other thin film cells)^5^,^11^ in cells made using solution-based synthesis methods^12^,^13^, in addition to other favourable properties, such as long carrier diffusion lengths^14^,^15^ and defect self-regulation^16^, the future development of high efficiency, easily processable modules looks promising. There are, however, still significant obstacles to overcome - particularly their recently revealed fundamental thermodynamic instability^17^ and the toxicity of lead^9^. However, recent work has shown that the environmental impact of lead leaching from a single broken module may not be high^18^, and that, in effect, lead’s ecotoxicity is in fact lower than that of tin^19^, which is the primary target of efforts made to develop stable lead-free cells^20^, which have much lower PCEs than their lead counterparts.

In this work, we intend to move beyond methylammonium and investigate another hybrid lead halide compound, recently discovered by Liu *et al*.: (Pb_2_I_6_) · (H_2_DPNDI) · (H_2_O) · (NMP) (DPNDI: N,N’-di(4-pyridyl)-1,4,5,8-napthalene diimide; NMP: N-methylpyrrolidin-2-one)^21^. The structure of the black crystal they produced is based around 1D [Pb_2_I_6_]^2−^ polyanions surrounded by an interwoven network of protonated DPNDI ligands, as seen in Fig. 1, both of which are linked through hydrogen bonding to each other and the solvent molecules. As [H_2_DPNDI]^2+^ is much larger than methylammonium, the dimensionality of the inorganic framework is reduced from the 3D perovskite structure to 1D lead iodide chains, a result consistent with the iodobismuthates^22^. The effect of bulkier organic cations has been recently examined in alkylammonium lead halides, with cells showing improved stability, however the lower connectivity of the lead iodide octahedra compared to CH_3_NH_3_PbX_3_ has lead to larger band gaps and low efficiencies^23^,^24^. Photoactive organic cations have been less well explored, with the only paper investigating tropylium lead iodide, and producing a band gap of 1.97 eV, which is outside of the range for high efficiency solar absorption^25^.

The lowering of dimensionality when compared to the methylammonium lead halides, and corresponding structure, is also consistent with a related hybrid lead iodide, MVPb_2_I_6_ (MV^2+^: N,N’-dimethyl-4,4’-bipyridinium, also known as methylviologen). Both exhibit the same distorted [Pb_2_I_6_]^2−^ unit, hypothesised to be due to the relatively strong dispersion interactions between the iodine atoms and the *π* bonding system on the organic linkers^21^,^26^. Equally, the lead iodide chains are separated by another layer of organic ligands to maximise the number of nearest neighbour iodine atoms to each *π* system. Of particular interest, however, was the observation of a photocurrent enabled by charge transfer between the organic and inorganic networks, allowed by the relative energies offset between the valence band of [Pb_2_I_6_]^2−^ and the methylviologen LUMO and confirmed by experiment and Density Functional Theory (DFT) calculations^27^,^28^. The resulting optical absorption band was observed between 2.0 and 2.6 eV, which is above the optimal absorption of 1.1–1.6 eV for an efficient solar cell^1^. With the protonated DPNDI ligand expected to have a lower energy LUMO than methylviologen, however, and the (Pb_2_I_6_) · (H_2_DPNDI) · (H_2_O) · (NMP) crystal observed to absorb across the entire visible spectrum^21^, the photovoltaic possibility of such an absorber is strongly worth investigating further. In this work, we will use ab initio DFT calculations to investigate this system and critically assess its suitability as a solar absorber.

## Methods

All calculations were performed using periodic DFT using the Vienna *ab initio* Simulation Package (VASP)^29^,^30^,^31^,^32^. Three different functionals were used to examine this system: PBEsol^33^, which revises the Generalised Gradient Approximation (GGA) PBE^34^ functional for solids; PBE0^35^, a hybrid density functional which encompasses 25% exact Hartree-Fock (HF) exchange, together with 75% exchange and the correlation energies from PBE^34^ and HSE06^36^, a screened modification to PBE0, which uses HF exchange only at short ranges. PBEsol has been shown to provide accurate lattice parameters for solid state systems, particularly hybrid halide perovskites^37^,^38^,^39^, while HSE06 gives both accurate lattice parameters and band energies in comparison with experiment^40^,^41^,^42^. The interactions between core and valence electrons in the system were described with the scalar relativistic projector-augmented wave (PAW) method^43^,^44^. In all examples, a cutoff energy of 450 eV and, with a unit cell containing 146 atoms and 11.361 × 13.250 × 15.572 Å in size, a Γ-centered 2 × 2 × 2 k-mesh were found to be sufficient. Calculations were converged once forces on each atom did not exceed 0.01 eV Å^−1^. For the pseudopotential treatment of Pb, 6s, 6p and 5d orbitals were considered valence.

In addition, while electrostatic forces hold the [H_2_DPNDI]^2+^ and [Pb_2_I_6_]^2−^ frameworks together, the solvent molecules in the crystal are bound solely by intramolecular dispersion forces and hydrogen bonding, which most GGA functionals are unable to correctly describe. To account for this, the D3 van der Waals correction method produced by Grimme *et al*.^45^ was used, and the results (denoted +VdW) compared with the uncorrected calculations. Finally, as Pb and I are both heavy atoms, the contribution of spin-orbit coupling to the electronic structure is significant, and, indeed, it has been shown that accounting for spin-orbit coupling is crucial to attain accurate energy values for hybrid halide perovskites^46^. As such, PBEsol calculations with a spin-orbit perturbation included were performed to assess the strength of this interaction in this system (denoted +SOC).

## Results and Discussion

Table 1 shows a comparison of the lattice parameters obtained from each of the relaxation calculations performed. Despite the lack of connectivity, PBEsol gives lattice parameters with the best correlation to experimental values given by Liu *et al*.^21^: 0.30% or less difference from experiment for each of the lattice parameters, and 0.80% or less for the cell angles - leading to a overestimation in cell volume of less than 1%. As such, the addition of the van der Waals correction (PBEsol + VdW) means that the lattice parameters were underestimated by 1–4%, as the networks are held closer together than expected. It appears that PBEsol accounts for some of the effects of dispersion forces on the structure, as shown by the improvement in cell volume (from 6.2% underestimation with PBEsol + VdW to 1.4%) on using PBE as the base functional (PBE + VdW), however even then the cell parameters still show greater variance from experiment than with PBEsol alone. Adding spin orbit coupling to PBEsol (PBEsol + SOC) results in negligible change in cell lengths compared to experiment (less than ±0.16%), and a small improvement in cell volume, but distortions of close to 1% in cell angles, while including both spin orbit coupling and the van der Waals correction results in a universal, but also negligble (no more than ±0.56%), improvement on PBEsol + VdW, and an even smaller cell volume. From this, the relaxed PBEsol unit cell was found to be suitable as the starting point for the HSE06 electronic calculations, as in the absence of thermal effects present in the experimental structure, some deviation is expected.

**Figure 2:**
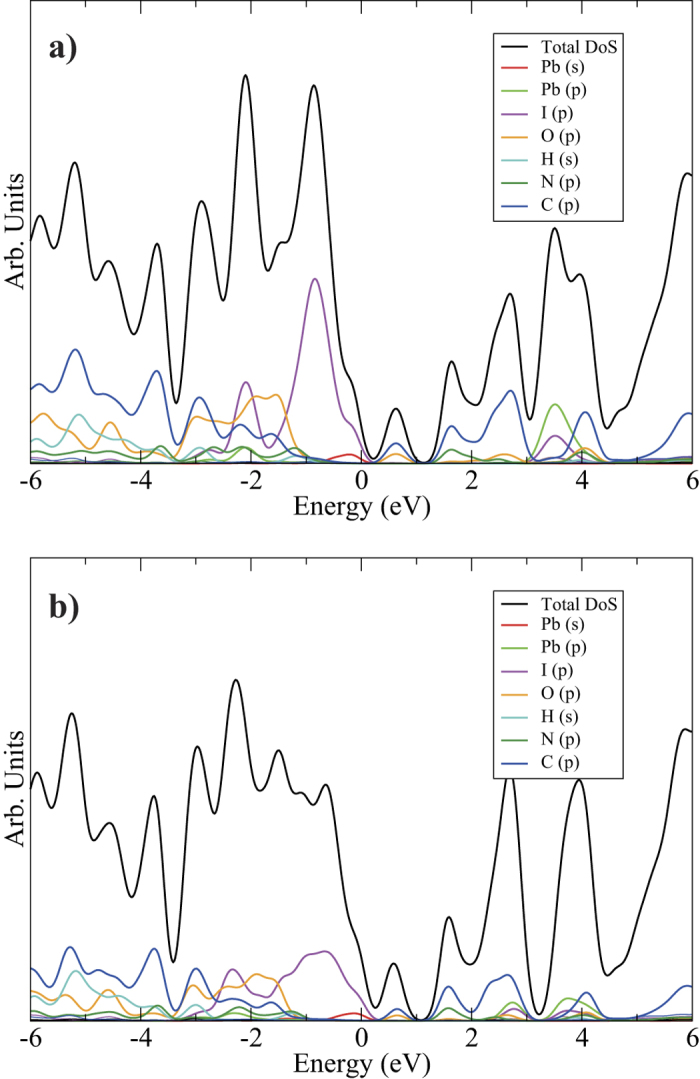
Total and Partial Density of States diagrams, using (**a**) PBEsol and (**b**) PBEsol + SOC; individual partial DoS are labelled in legends, Energy = 0 eV is set to valence band maximum and gaussian smearing of 0.2 eV was used.

**Figure 3:**
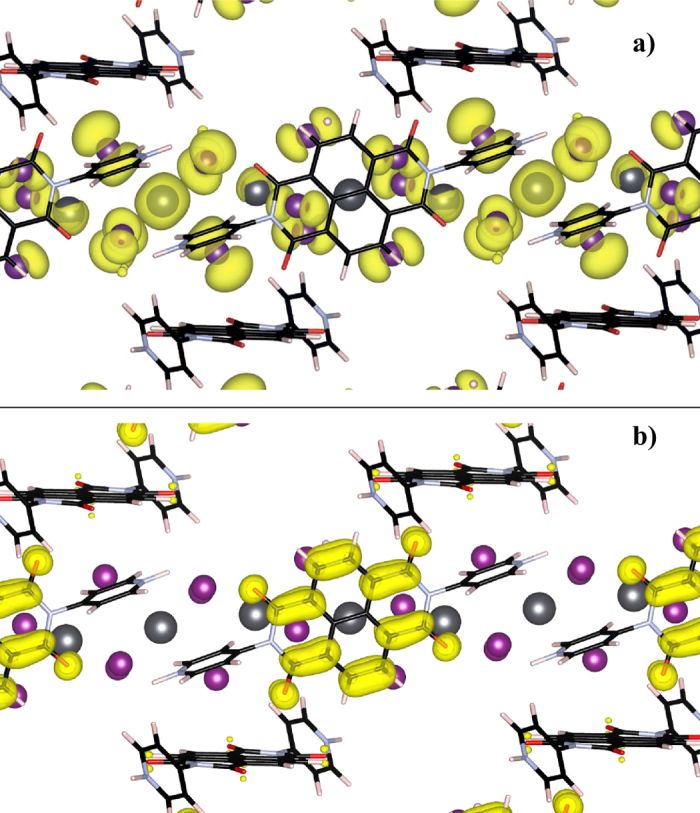
Plots of partial charge density at band edges: (**a**) Valence band; (**b**) Conduction band; viewed along the a crystallographic axis, with electron density marked in yellow.

Figure 2 displays the calculated Density of States (DOS) diagrams, decomposed into total and partial density of states, for the PBEsol and PBEsol + SOC relaxations. Most significantly, both show that the top of the valence band is dominated by the inorganic [Pb_2_I_6_]^2−^ network (lead *s* states and iodine *p*), while the conduction band is wholly situated on the organic ligands - predominantly carbon, oxygen and nitrogen *p* states. This suggests that this compound exhibits spatial charge separation on photoirraditation: in a direct transition, electrons will transfer onto the organic network, while the holes remain on the lead iodide nanowires. This observation is supported by plotting the partial charge density maps for the valence band maximum (VBM) and conduction band minimum (CBM) seen in Fig. 3, which show that the valence band is distinctly comprised of the lead s and iodine p orbitals, while the conduction band sits firmly on the organic ligand. A partial charge calculation on a 30 Å × 30 Å × 30 Å box containing a single DPNDI molecule confirmed that the conduction band minimum shown matches the LUMO of DPNDI, as predicted by Liu *et al*.^21^. In addition, charge separation of electrons onto the organic DPNDI ligand correlates with the observed electron spin resonance pattern of a DPNDI radical. Otherwise, there is little qualitative difference between PBEsol and PBEsol + SOC and the spin-orbit effect on the relative energy levels, including the Pb and I *p* states, is small. The VdW corrected calculations showed very similar DOS plots to the corresponding uncorrected calculation in Fig. 2 and are shown in Supplementary Figs S1–S3.

**Figure 4:**
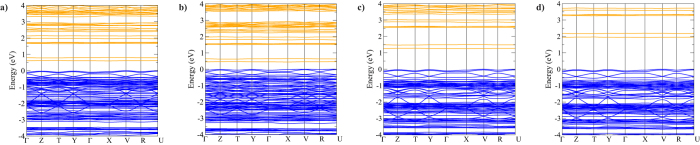
Band structure diagrams, using (**a**) PBEsol, (**b**) PBEsol + SOC, (**c**) HSE06, and (**d**) PBE0; valence band marked in blue, conduction band marked in orange, Energy = 0 eV is set to valence band maximum.

To further assess this compound’s capability as an absorber, the electronic band structure was calculated with each of the PBEsol-based methods, and in addition using hybrid functionals PBE0 and HSE06, using the relaxed PBEsol structure. The resulting diagrams are shown in Fig. 4. The D3 correction was observed to have little energetic effect on the band structures and so those diagrams are shown in Supplementary Figs S4–S6. Again, there is little qualitative difference between the diagrams other than the doubled number of bands in the non-collinear spin-orbit coupled calculation, indicating a consistent description of the electronic structure. In all diagrams, the organic-based conduction band shows only very small dispersion, indicating a high electron effective mass; these were calculated using the band curvatures from the HSE06 result as 8.33 m_0_ and 1.96 m_0_ for the conduction and valence band respectively. These values are much higher than the equivalents for methylammonium lead iodide (MAPI) obtained using DFT (0.23 m_0_ and 0.29 m_0_ respectively)^47^, the difference likely due to the lower connectivity of [Pb_2_I_6_]^2−^ compared to the lead iodide perovskite, which is enforced by the larger organic cation hindering the orbital overlap. However, the charge separation from the lead and iodide-based valence band may strongly reduce potential recombination losses expected from low conductivity^48^. Additionally, it is clear that there are significant differences in the predicted indirect band gap energies between the methods used, also displayed in Table 2. PBEsol gives a small fundamental band gap of 0.60 eV, which is reduced further on introduction of spin-orbit effects to 0.44 eV. These values would suggest a band gap too low for effective use in a photovoltaic cell, yet GGA DFT methods are known to underestimate band gaps, sometimes severely^49^. Moving to the hybrid functionals opens up the band gaps to within the ideal 1.0–1.6 eV range: HSE06 gives an indirect fundamental band gap of 1.26 eV while PBE0 gives 1.94 eV. The large difference between these two functionals is likely due to the significant effect of the screening on the orbital energies – and, as mentioned above, HSE06 has been shown to give more accurate results for solid semiconductors^40^,^41^,^42^. We would expect a similarly small spin-orbit effect with these two hybrid functionals as with PBEsol, and a decrease in band gap of 0.16 eV would maintain a hypothetical HSE06 + SOC band gap within the target range.

To further investigate the optical behaviour of this system, the optical absorption was calculated; however, due to the large size of the system, such a calculation was only possible at the PBEsol level. The resulting plot of the attenuation coefficient *α* against energy is shown in Supplementary Fig. S7. From this, it is predicted that the compound should demonstrate moderately strong absorption (~10^4^ cm^−1^) just above the fundamental band gap, which, in comparison to the HSE06 result, would mean absorption within the optimal 1.0–1.5 eV range. This supports the experimental absorption measurements by Liu *et al*. that demonstrated that the compound does absorb within the visible electromagnetic range^21^.

In addition, the thermodynamic stability of the compound was assessed in comparison to MAPI’s decomposition pathway. As noted above, theoretical study by Zhang *et al*.^17^ and Ganose *et al*.^50^ has shown that MAPI is expected to be thermodynamically unstable, with a reaction enthalpy of −0.09 eV, compared to CH_3_NH_3_I and PbI_2_, the commonly observed byproducts of MAPI degradation. As such, we have probed the thermodynamics of the analogous reaction for this compound:


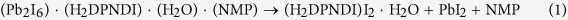


Utilising structures from the literature^21^,^51^,^52^, and relaxing them with the PBEsol functional, the enthalpy of reaction 1 was found to be endothermic, with Δ_*r*_*H* = +2.03 eV, indicating that this compound does not have a similar intrinsic instability, and should be more resistant to decomposition than MAPI.

The structure-property relationships of similar dense hybrid inorganic-organic materials are still relatively unexplored, but it is evident from the study of this compound, and those on MVPb_2_I_6_, that further exploration of photoactive organic cations in hybrid photovoltaic materials could lead to very useful device properties like efficient charge separation and suitable electronic band gaps, together with improved stability.

## Conclusion

The crystal and electronic structures of the hybrid inorganic-organic lead iodide (Pb_2_I_6_) · (H_2_DPNDI) · (H_2_O) · (NMP) have been examined using a variety of DFT methods, with a focus on assessing its capability as a photovoltaic material. The compound was shown to have an indirect band gap of 1.26 eV with the hybrid HSE06 functional, and spatial valence band and conduction band separation due to the inclusion of a photoactive organic cation, which may lead to the reduction of recombination losses. This comes at the cost of the large size of the cation leading to lower [Pb_2_I_6_]^2−^ connectivity and corresponding high effective carrier masses. It is clear that photoactive organic cations, like DPNDI, can allow suitable band gaps for photovoltaic devices, hence further effort into producing such hybrid materials without sacrificing connectivity, and resultant mobility, is highly recommended.

## Additional Information

**How to cite this article**: Savory, C. N. *et al*. Spatial Electron-hole Separation in a One Dimensional Hybrid Organic-Inorganic Lead Iodide. *Sci. Rep*. **6**, 20626; doi: 10.1038/srep20626 (2016).

## Supplementary Material

Supplementary Information

## Figures and Tables

**Figure 1:**
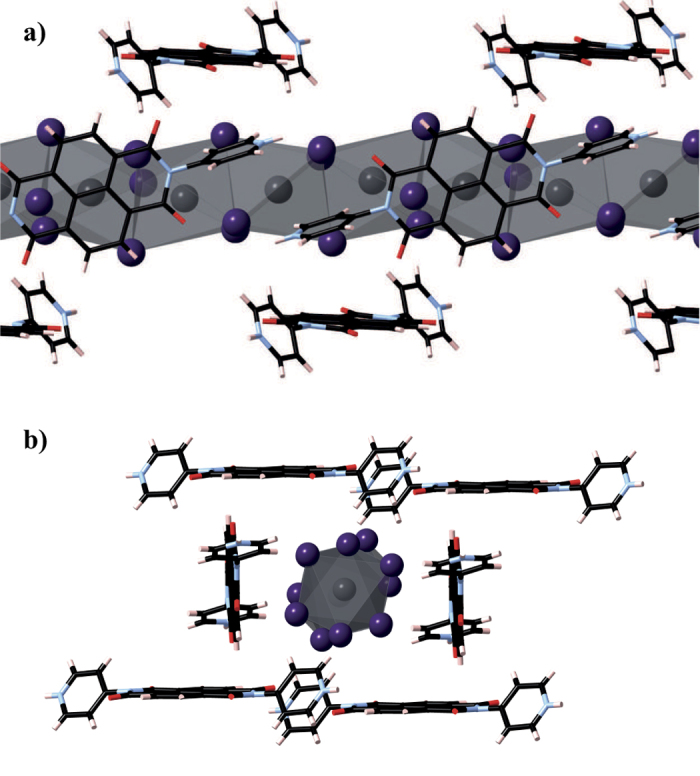
(Pb_2_I_6_) · (H_2_DPNDI) · (H_2_O) · (NMP), with solvent molecules removed for clarity and viewed along: (**a**) the a crystallographic axis, and (**b**) a single [Pb_2_I_6_]^2−^ nanowire, showing the DPNDI network. Carbon atoms are marked in black, hydrogen in pink, oxygen in red, nitrogen in blue, iodine in purple and lead in grey.

**Table 1:** Calculated lattice parameters of (Pb_2_I_6_) · (H_2_DPNDI) · (H_2_O) · (NMP), with experimental error in brackets.

	a/Å	b/Å	c/Å	*α*/°	*β*/°	*γ*/°	Volume/Å^3^
PBEsol	11.370	13.260	15.619	66.91	78.72	75.15	2085.60
PBEsol + VdW	11.054	12.728	15.325	68.43	77.26	75.27	1937.28
PBEsol + SOC	11.370	13.236	15.596	66.97	78.84	75.86	2082.05
PBEsol + VdW + SOC	11.090	12.795	15.360	68.19	77.35	75.15	1919.92
PBE + VdW	11.269	13.077	15.585	67.69	77.83	75.83	2037.92
Experiment^21^	11.361(2)	13.250(3)	15.572(3)	66.81(3)	78.10(3)	74.92(3)	2066.24

**Table 2:** Calculated band gap values.

Functional	Indirect band gap/eV	Direct band gap at VBM/eV
PBEsol	0.60	0.62
PBEsol + SOC	0.44	0.47
HSE06	1.26	1.27
PBE0	1.94	1.95
