# Supplementary material for: Spatial Electron-hole Separation in a One Dimensional Hybrid Organic–Inorganic Lead Iodide
Source: Sci Rep. 2016 Feb 9;6:20626. doi: 10.1038/srep20626 (PMC4746642; doi:10.1038/srep20626)
Supplement: Supplementary Information [file srep20626-s1.pdf]

Supplementary Information: Spatial Electron-hole Separation in a One Dimensional Hybrid Organic–Inorganic Lead Iodide

Christopher N. Savory,[†] Hugo Bronstein,[‡] Robert Palgrave,[‡] and David O. Scanlon[*,†,¶]

[†]*University College London, Kathleen Lonsdale Materials Chemistry, Department of Chemistry, 20 Gordon Street, London WC1H 0AJ, UK*

[‡]*University College London, Department of Chemistry, London WC1H 0AJ, UK*

[¶]*Diamond Light Source Ltd., Diamond House, Harwell Science and Innovation Campus, Didcot, Oxfordshire OX11 0DE, UK*

E-mail: d.scanlon@ucl.ac.uk

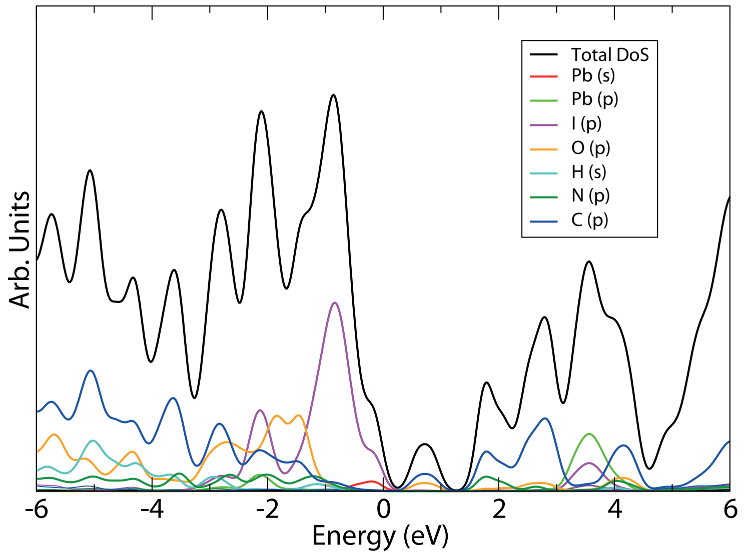

Supplementary Figure S 1: Total and Partial Density of States diagram, using PBEsol+VdW; individual partial DoS are labelled in legends, Energy = 0 eV is set to valence band maximum

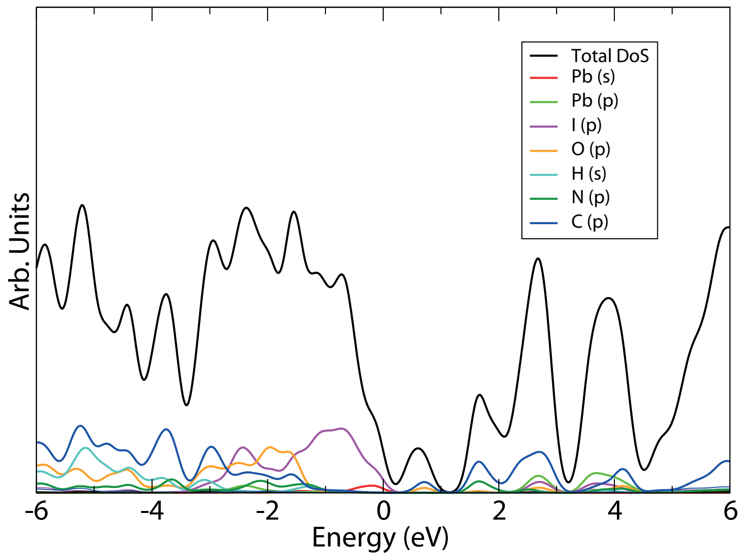

Supplementary Figure S 2: Total and Partial Density of States diagram, using PBEsol+VdW+SOC; individual partial DoS are labelled in legends, Energy = 0 eV is set to valence band maximum

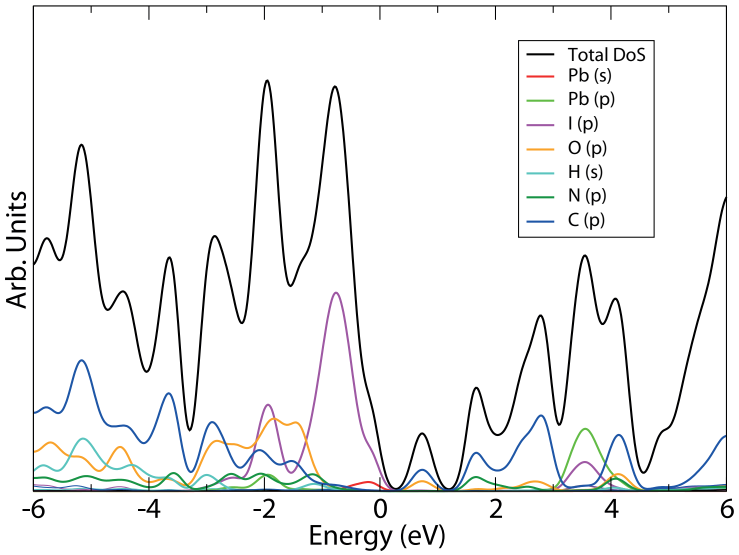

Supplementary Figure S 3: Total and Partial Density of States diagram, using PBE+VdW; individual partial DoS are labelled in legends, Energy = 0 eV is set to valence band maximum

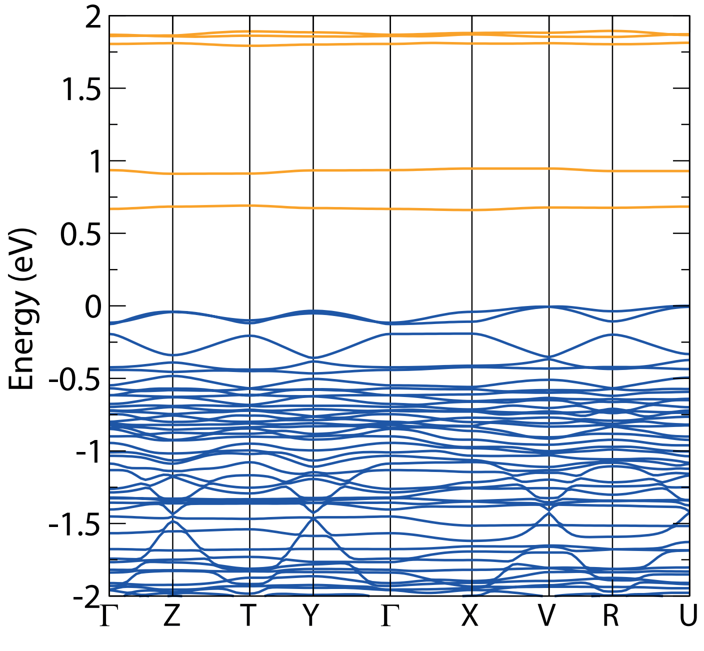

Supplementary Figure S 4: Band structure diagram, using PBEsol+VdW; valence band marked in blue, conduction band marked in orange, Energy = 0 eV is set to valence band maximum

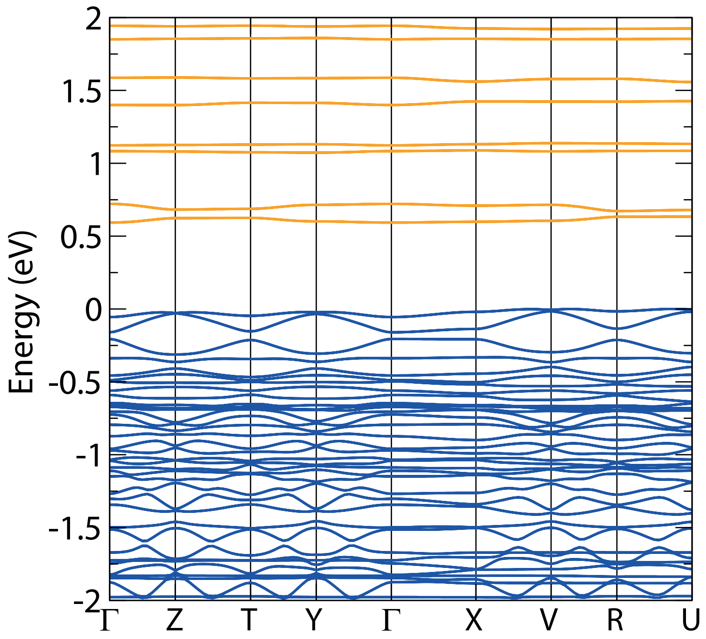

Supplementary Figure S 5: Band structure diagram, using PBEsol+SOC+VdW; valence band marked in blue, conduction band marked in orange, Energy = 0 eV is set to valence band maximum

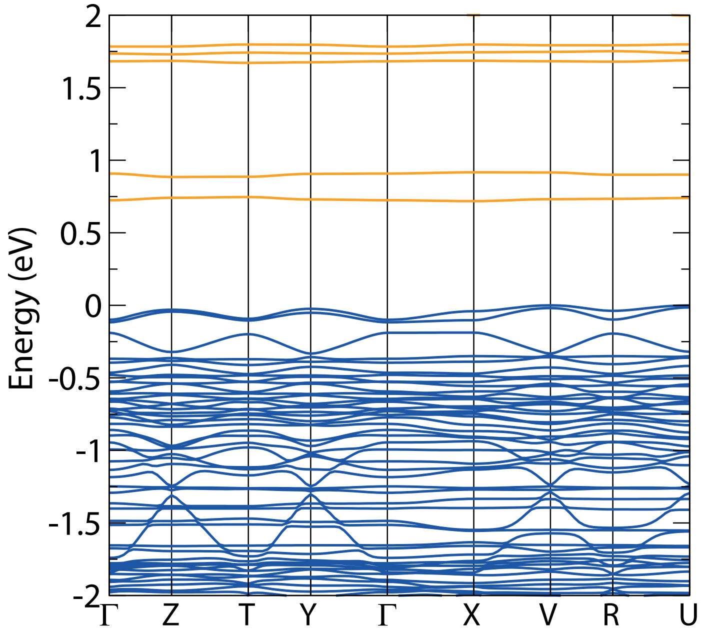

Supplementary Figure S 6: Band structure diagram, using PBE+VdW; valence band marked in blue, conduction band marked in orange, Energy = 0 eV is set to valence band maximum

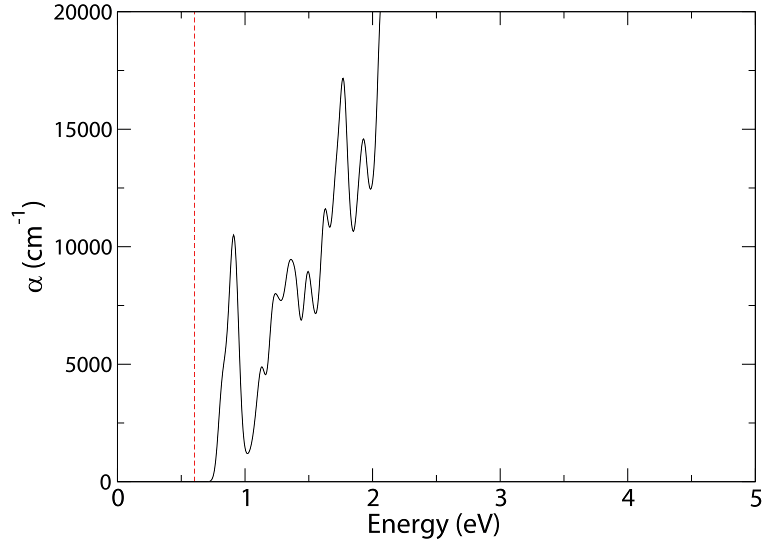

Supplementary Figure S 7: Plot of optical absorption using PBEsol, with a gaussian smearing of 0.025eV; fundamental band gap is marked with a dashed line

VASP CONTCAR file: PBEsol

(Pb2I6).(H2DPNDI).(H2O).(NMP) PBEsol

1.00000000000000

11.3699320415362664 -0.0700664176839490 -0.0362500987695058

3.3703328360904314 12.8249589172267964 0.0046184668813145

3.1342204387400256 5.5056884862186557 14.2761852262716449

Pb I O H N C

4 12 12 50 10 58

Direct

0.9999828988039638	0.5000332585138167	0.4998870123032901
0.0103500758701500	0.7684367896046496	0.2625730622647708
0.9896497177817736	0.2315565854062100	0.7374319798363587
0.9999980655680574	0.9999100930327742	0.0001245860397674
0.1888527712040329	0.3801470844932311	0.6541204203163815
0.8111122226610448	0.6198625294224485	0.3458666050559032

0.2087201337111821	0.5404765063541603	0.3250817093377663
0.7912666361412946	0.4595306033578339	0.6748408893227520
0.0100520300189757	0.7499833425520350	0.4761898278085326
0.9898965734819782	0.2500316381413867	0.5237441276163608
0.1696655739937754	0.9685248358852334	0.1574737251488543
0.8303086180922747	0.0314995246998180	0.8425410926200314
0.2138377545672014	0.0287876281981738	0.8304105539763924
0.7862115698861913	0.9709394412996915	0.1695958394960613
0.0608075666920556	0.7338901689237218	0.0676387671204353
0.9392267886783756	0.2660346465295689	0.9323687597204184
0.6797180752386041	0.2644566833278859	0.2298437577470338
0.3202869054324324	0.7355458322494997	0.7701558730841711
0.1489337733151004	0.4525100949288827	0.9990540265761538
0.8510663116896495	0.5474985254280753	0.0009517199864959
0.5938048500948554	0.5150750044716403	0.2214556418415157
0.4061978038656093	0.4849309048540462	0.7785464281346535
0.5549136867499413	0.1757025900838443	0.7029517762664810
0.4450888492362424	0.8243039911052321	0.2970483820112193
0.2648820738515809	0.1715776265730327	0.3367915526980241
0.7351205494496824	0.8284271826032139	0.6632087860504683
0.4288435847642091	0.0488584264638448	0.2029623019214526
0.5711554592497095	0.9511459918430560	0.7970391446868703
0.3410215381923791	0.0474040851677415	0.2176249126938998
0.6589782348294762	0.9526010322308665	0.7823839290357100
0.5336361917768997	0.0309213751460007	0.7695229437500899
0.4663622762634247	0.9690833294855992	0.2304782743726648
0.1497952519668289	0.2573188563237778	0.2949660584336868

0.8502061674521713	0.7426851006895561	0.7050374277680831
0.4567664047511002	0.1038148270795745	0.0982257724351356
0.5432321562311415	0.8961928351558939	0.9017735295184721
0.0881968898184624	0.1342752436369707	0.2515413230447052
0.9118050855507462	0.8657303281692208	0.7484615501495924
0.0521120724971027	0.4511811260970262	0.2586345163871826
0.9478910794886630	0.5488218332121519	0.7413658452123073
0.1317896473480431	0.4752540420115210	0.8310565045722100
0.8682118127711078	0.5247500228079716	0.1689444172057009
0.9057657769069607	0.1940604411913895	0.1619657826667975
0.0942379100290509	0.8059491568395742	0.8380319467731923
0.2746953157299075	0.3326753484227609	0.1301477727073248
0.7253072093658020	0.6673313489618593	0.8698536364389895
0.4651632903249592	0.2615720277990263	0.2114261202605405
0.5348386224728898	0.7384354843725234	0.7885757156462390
0.6377566827767680	0.1772696155768898	0.0264796876647537
0.3622425085481140	0.8227413994782893	0.9735175767476605
0.3401719257693259	0.7081247298670519	0.1478468761614025
0.6598255530212001	0.2918813809327503	0.8521547254069120
0.3070745626194338	0.2312806174602144	0.8393040261729823
0.6929229750895729	0.7687218867194971	0.1606964178192243
0.2976161187739805	0.1220057349536958	0.0146835143270536
0.7023840067804343	0.8779983528628890	0.9853190175724151
0.6311120902588954	0.3315007457859096	0.3589605596379002
0.3688893096663222	0.6685048192422656	0.6410421041911434
0.6208224035712533	0.2118595379946413	0.5316010733681225
0.3791804188585814	0.7881459505486674	0.4684017906089153

0.6585581140151007	0.0650541644610385	0.3881476797160204
0.3414424398436111	0.9349527911702964	0.6118545945335967
0.2123153218906282	0.0387711120275966	0.6231689287337332
0.7876857668856161	0.9612334786315486	0.3768343573103081
0.3540086967768943	0.1492726857430355	0.6081519878576884
0.6459933756518197	0.8507336140872042	0.3918501128551526
0.4757319301441711	0.0630738996816760	0.5641934953270749
0.5242688137884812	0.9369323802324772	0.4358079088437279
0.4498138102742288	0.2022941480464269	0.4115115501907880
0.5501862285332493	0.7977125066264605	0.5884905181718523
0.3090913781604314	0.2710461533677773	0.4526483937188672
0.6909094361793180	0.7289593492592559	0.5473544217771931
0.1075506849229981	0.0369741042765881	0.4384581238801033
0.8924495762753111	0.9630329628398115	0.5615448418135358
0.8459279258240144	0.0775153329838645	0.4581156304002789
0.1540736843284662	0.9224919109348519	0.5418867819763022
0.7653569490368568	0.0703034577626838	0.5703259527366313
0.2346457542815727	0.9297030830226944	0.4296756284769501
0.0718191209596242	0.2916324008918778	0.2565872815783479
0.9281837627809324	0.7083723993120685	0.7434133970872594
0.7685676896100091	0.4008979891815940	0.1114660268298238
0.2314340374516490	0.5991075998817124	0.8885356101273771
0.4668550714607846	0.1499494251341744	0.0223795823636905
0.5331447581589188	0.8500544116201283	0.9776217793572144
0.5085347066261221	0.6660540473606389	0.2624007235972812
0.4914621480952377	0.3339505059128030	0.7376017045613708
0.2654662083719117	0.0414603648903906	0.4859073136688750

0.7345345078033816	0.9585461326403859	0.5140941989167800
0.0345989261104762	0.2193097442255691	0.2317664460330846
0.9654037409730378	0.7806956955624571	0.7682346167656959
0.0152367448112258	0.3987607579254586	0.2349674007661946
0.9847645798162006	0.6012427277589438	0.7650339023729842
0.0853952673197256	0.5621070340301273	0.8144760202971284
0.9146051370450294	0.4378966276377909	0.1855266868934820
0.8738151823688582	0.3641089947585172	0.1597638526879948
0.1261856358272198	0.6358957638317762	0.8402377286033769
0.9346172405329014	0.2532069049843102	0.1826282934497883
0.0653855370163328	0.7467989701794053	0.8173729164416770
0.6708121065858776	0.3438288784022276	0.1552171014215205
0.3291911573444679	0.6561768575345326	0.8447833502859510
0.5596626316519533	0.3860307093335251	0.1070063998190847
0.4403407726307975	0.6139747613069346	0.8929944073936866
0.5540346455483842	0.4798562974247531	0.0226570294050035
0.4459682056014103	0.5201487400390548	0.9773441051812526
0.3464695878466912	0.4647256687575236	0.0174356131130295
0.6535319005900391	0.5352807537685464	0.9825652880957065
0.7651512890976022	0.4981782743458467	0.0297227194002261
0.2348494196199340	0.5018302698264065	0.9702791668852058
0.3536540851808709	0.3734487278662826	0.1007427815549562
0.6463486502612668	0.6265575841932360	0.8992582745389583
0.4599232168816627	0.3339295181306596	0.1458845257746759
0.5400793229968031	0.6660772813476328	0.8541167452012743
0.4327374226595779	0.8061137381266690	0.0186728177700530
0.5672619630234905	0.1938902493399723	0.9813278177761973

0.4212640995501005	0.7450172007449964	0.1138389980056829
0.5787342003548801	0.2549873106775493	0.8861620881041219
0.5158560979994746	0.7315114593665015	0.1643956059221594
0.4841413111012187	0.2684913982276100	0.8356062287422859
0.3813409181488225	0.2214740296824047	0.8789857332134190
0.6186574514029672	0.7785255039685879	0.1210154981051872
0.6248884062637288	0.8381294064053790	0.0256843137081901
0.3751116824169429	0.1618719905474109	0.9743171011748757
0.5304413850220726	0.2786084150369348	0.6745039018018772
0.4695582635440090	0.7213968720240800	0.3254980759327992
0.5399175015775270	0.3500219005550136	0.5750576686037476
0.4600827567445975	0.6499843567382015	0.4249438998143233
0.5021780313316739	0.5335036243055384	0.4517996139711329
0.4978220022722155	0.4665021743608406	0.5482021237608521
0.5499159469094863	0.4824060977422917	0.3849843077710986
0.4500845005113163	0.5175991013719070	0.6150178873024146
0.5535575649525484	0.5511569329184383	0.2849971921083423
0.4464432338367104	0.4488468608320915	0.7150059737900278
0.5935240916977023	0.3682117655721129	0.4127124222154279
0.4064772970278483	0.6317935440694384	0.5872902723256601
0.5880072085058714	0.3021452141208485	0.5080606505007879
0.4119950235421399	0.6978601753367926	0.4919420329204272
0.2969023742429400	0.0225403515960991	0.5777152533667689
0.7030978557066661	0.9774662757549564	0.4222870111786463
0.3800417644865064	0.1066108338725726	0.5574350721563661
0.6199588032279735	0.8933955306512331	0.4425670756000599
0.3634796971670511	0.1879034612068082	0.4568323220312820

0.6365204298344977	0.8121022674669334	0.5431697718520851
0.2938041909695528	0.1352017549081239	0.4186493952096342
0.7061963314766260	0.8648038206951796	0.5813524936773433
0.8131312807893067	0.0207124202337070	0.5268372741715410
0.1868697503212431	0.9792939488191692	0.4731644038859883
